# JAK/STAT Signaling: Molecular Targets, Therapeutic Opportunities, and Limitations of Targeted Inhibitions in Solid Malignancies

**DOI:** 10.3389/fphar.2022.821344

**Published:** 2022-03-24

**Authors:** Bilal Rah, Rafiq A Rather, Gh Rasool Bhat, Abdul Basit Baba, Ifra Mushtaq, Muzamil Farooq, Tahira Yousuf, Sadaf B Dar, Sabra Parveen, Rukhsana Hassan, Fozia Mohammad, Iqbal Qassim, Abida Bhat, Shazia Ali, Mahrukh Hamid Zargar, Dil Afroze

**Affiliations:** Advanced Centre for Human Genetics, Sher-I-Kashmir Institute of Medical Sciences, Srinagar, India

**Keywords:** solid tumors, signaling, molecular targets, therapeutic opportunities, inhibitors

## Abstract

JAK/STAT signaling pathway is one of the important regulatory signaling cascades for the myriad of cellular processes initiated by various types of ligands such as growth factors, hormones, and cytokines. The physiological processes regulated by JAK/STAT signaling are immune regulation, cell proliferation, cell survival, apoptosis and hematopoiesis of myeloid and non-myeloid cells. Dysregulation of JAK/STAT signaling is reported in various immunological disorders, hematological and other solid malignancies through various oncogenic activation mutations in receptors, downstream mediators, and associated transcriptional factors such as STATs. STATs typically have a dual role when explored in the context of cancer. While several members of the STAT family are involved in malignancies, however, a few members which include STAT3 and STAT5 are linked to tumor initiation and progression. Other STAT members such as STAT1 and STAT2 are pivotal for antitumor defense and maintenance of an effective and long-term immune response through evolutionarily conserved programs. The effects of JAK/STAT signaling and the persistent activation of STATs in tumor cell survival; proliferation and invasion have made the JAK/STAT pathway an ideal target for drug development and cancer therapy. Therefore, understanding the intricate JAK/STAT signaling in the pathogenesis of solid malignancies needs extensive research. A better understanding of the functionally redundant roles of JAKs and STATs may provide a rationale for improving existing cancer therapies which have deleterious effects on normal cells and to identifying novel targets for therapeutic intervention in solid malignancies.

## Introduction

**FIGURE 1:**
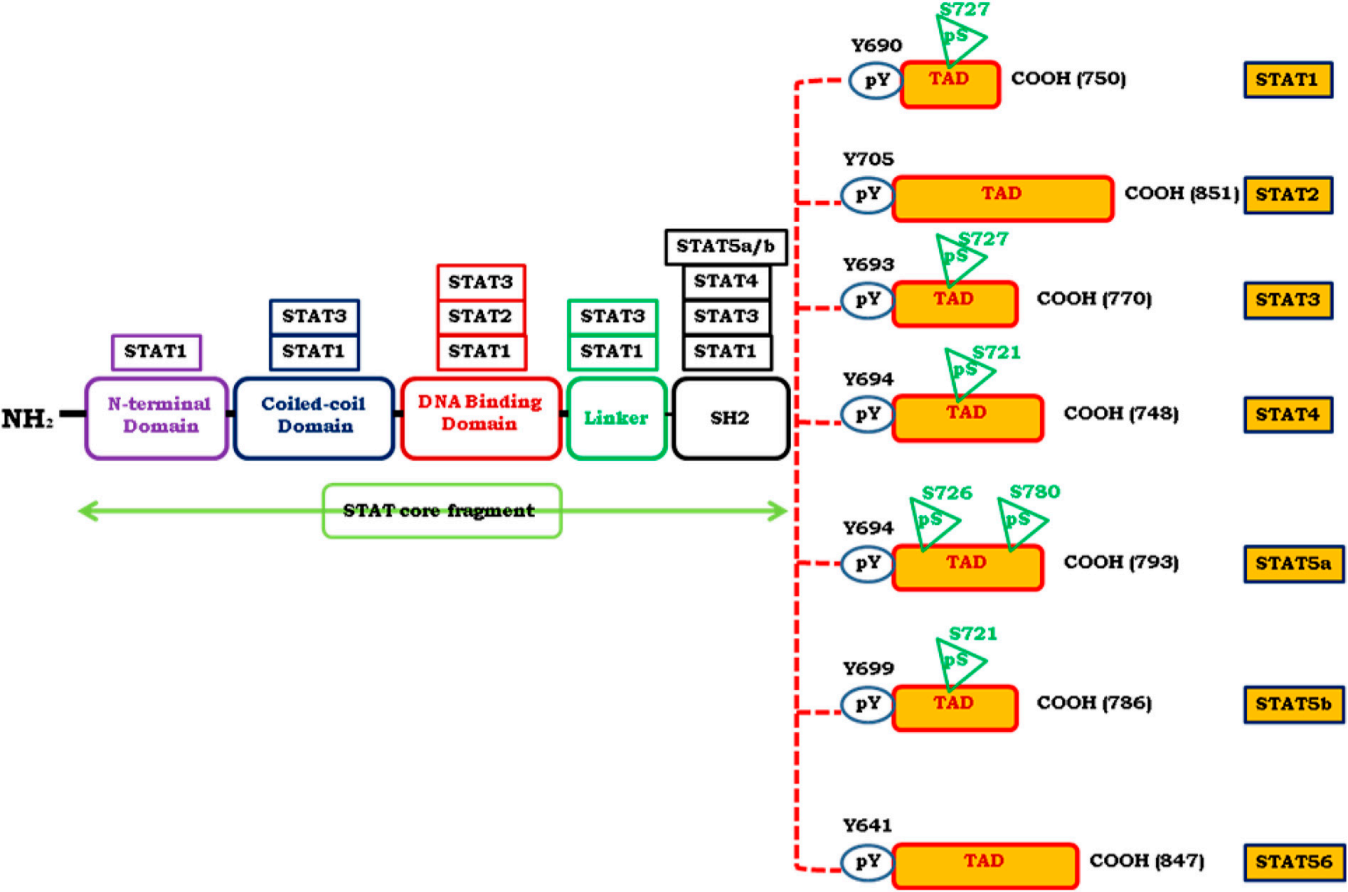
Represents the structural and functional composition of various conserved regions of STAT proteins. STAT proteins composed of N-terminal domain, coiled-coil domain, DNA binding domain, a linker domain, Src-homology 2 (SH2) domain and a C-terminal transcriptional activation domain (TAD). TAD possesses tyrosine phosphorylation (pY) and serine phosphorylation (pS) sites which are essential for STAT activation. Location of pS and pY are depicted in figure. The length of amino acid chain defined by the carboxylic acid (COOH) group is indicated on the right side of each STAT member.

**FIGURE 2:**
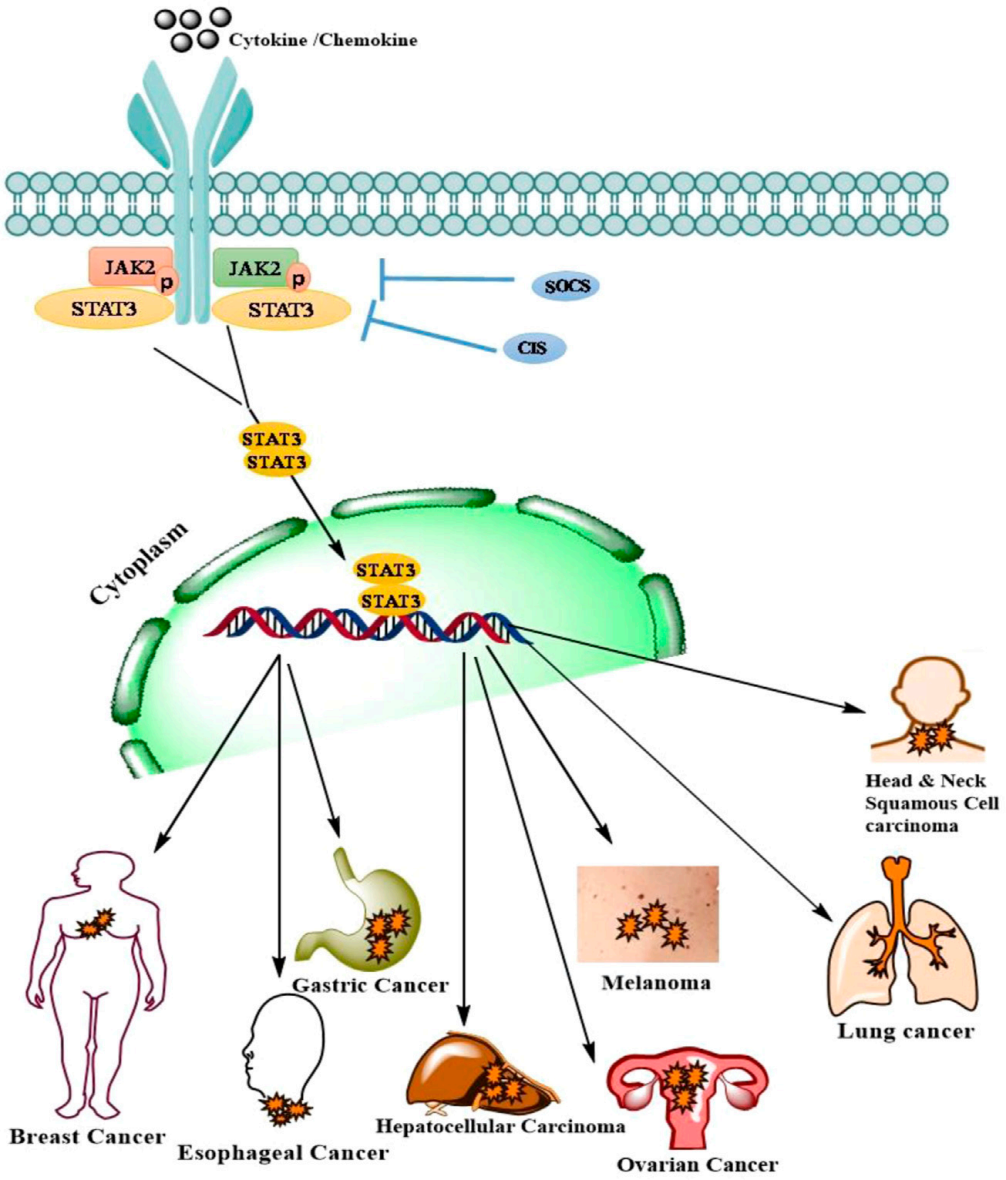
Represents the signaling mechanism of JAK/STAT and its contribution in the pathogenesis of various solid malignancies. JAK/STAT signaling in Melanoma.

An intracellular signaling pathway is critical in regulating the cellular fate and modulates phenotypic modifications. JAK/STAT signaling is one such pathway that regulates embryonic development, stem cell maintenance, hematopoiesis and, inflammatory response (Bowman et al., 2018). JAK/STAT is a signal transduction pathway, which transmits the extracellular information or signals through a transmembrane protein, called Janus kinase (JAK). The JAK further directs the signal to an intracellular environment by phosphorylating the transcription factor known as STATs, which translocates into the nucleus to target the promoter region of a gene to regulate the mechanism of transcription (Fiebelkow et al., 2021). The evolutionarily conserved pathway in all eukaryotes, JAK/STAT is a principal signal transduction pathway in mammals for cytokines and growth factors (O’Shea et al., 2015). Structurally there are four members of the JAK family i.e., JAK1, JAK2, JAK3, Tyk2, and seven members of STAT, i.e., STAT1, STAT2, STAT3, STAT4, STAT5A, STAT5B, and STAT6 in mammals (Abroun et al., 2015; Alunno et al., 2019) (Figure 1). As the ligands bind to the cognate receptor, the two JAK’s come closer and allow *trans*-phosphorylation of both the receptors as well as STATs at their conserved tyrosine residue present near to the C-terminal region (Morris et al., 2018). The phosphorylation of tyrosine at the C-terminal region is responsible for the dimerization of STATs which further enhances the interaction of a conserved domain called as SH2 domain (Morris et al., 2018). STAT is a transcription factor that resides in the cytoplasm. The phosphorylated STATs then enter the nucleus from the cytoplasm through a mediator called importin α-5 and Ran nuclear import (Seif et al., 2017). These dimerized STATs then bind to the particular regulatory sequence for the activation or repression of the targeted genes (Seif et al., 2017) (Figure 2). Thus, JAK/STAT is a signal transduction pathway that converts the extracellular signals into the transcriptional message thereby regulating physiological processes. Aberrant activation of intracellular signaling pathways confers malignant phenotype to genetically and metabolically altered cells (Flavahan et al., 2017). Many of these alterations occur in signaling pathways that control cell growth and division, cell death, cell fate, cell motility, tumor microenvironment, angiogenesis, and inflammation (Jing et al., 2019). Besides regulating physiological processes, JAK/STAT signaling cascade is implicated in various pathophysiological disorders including malignancies by altering the JAK/STAT signaling. In solid malignancies, these alterations characteristically support progression from a relatively benign group of proliferating cells (hyperplasia) to a mass of cells with abnormal morphology (dysplasia), cytological appearance, cellular organization, genomic integrity, and metabolic state (Gale et al., 2016). As a result of the structural and functional complexity of solid tumors, cancer therapies exhibit variable responses in distinct patients and cancer types (Cabrera et al., 2015). Although, the number of signaling pathways are deregulated in malignancies, however; besides being invariably activated in hematological malignancies, JAK/STAT signaling is, altered in many solid tumors and showed deregulated activation (Joshi et al., 2015). STATs are the effective downstream mediators of the JAK/STAT signaling cascade. STATs regulate the expression of a wide variety of genes both positively and negatively, identification of many of them is still imprecisely determined and warrants further investigation (Stark et al., 2018). The combination of STATs and the tissue in which they function at a given time often determines the subset of genes they control, which further contributes to the challenge of target gene identification. Constitutive phosphorylation of STAT1, STAT3, and STAT5 has been detected in many tumor cell models (Bellucci et al., 2015). The microarray-based expression analysis is used to comprehensively identify STAT target genes. Although STATs contributes to a malignant phenotype by regulating genes involved in cellular proliferation, survival, differentiation, angiogenesis, and invasion (Stark et al., 2018). However, how STATs execute these biological functions is critical for understanding the pathophysiology of solid tumors, signifying the need to understand the more precise role of JAK/STAT pathway in the pathobiology of solid malignancies. Despite this, some differences in STAT targets among cell types may be due to epigenetic variation among cells, which includes altered histone modifications or DNA methylation (Biswas and Rao, 2017; Garg et al., 2018). Thus, there is a need to identify the new target genes of STATs to fully understand how JAK/STATs can be used for the therapeutic intervention of solid tumors. Indeed, solid tumors contain aberrantly activated transcription factors—either through mutation of the transcription factor itself or through mutation of upstream signaling cascades leading to its activation (Qureshy et al., 2020; Gilmore, 2021). Therefore, understanding the genes regulated by STATs may provide insights into the pathophysiology of solid tumors in which they are inappropriately activated and may unravel novel targets for therapeutic intervention. Targeting intracellular signaling pathways has been a productive strategy for drug development, with several drugs acting on JAK-STAT signaling already in use and many more are being developed. In this review, we provide a comprehensive review of the role of the JAK/STAT pathway in solid tumors, clinical evidence of targeted agents, and also discuss the therapeutic intervention of JAK/STAT in solid tumors. The knowledge that we gathered in this review can be used in the strategic design of future researches and in the development novel targets and therapeutic modalities.

### JAK/STAT Signaling in Melanoma

Several intracellular and extracellular signals are transduced by the JAK/STAT pathway to regulate growth, proliferation, differentiation, development, and homeostasis (Dodington et al., 2018). Under normal physiological conditions, JAK/STAT pathway is activated to induce the secretion of a wide variety of growth factors, cytokines, interleukins, and hormones (Kraemer et al., 2020). Thus, the activation of JAK/STAT signaling may lead to increased proliferation, differentiation, migration, and cell death (Gu et al., 2016). However, aberrant activation of this pathway in skin cells leads to unregulated cell proliferation and cancer development (Khan A. Q. et al., 2019; de Araújo et al., 2019). Consequently, this signaling cascade plays an important role in the development of melanoma, a highly lethal and therapeutically resistant form of skin cancer (Lazăr et al., 2020). At the molecular level, this pathway is relatively uncomplicated. A wide range of ligands (cytokines, growth factors, hormones) and their cognate receptors participate in the activation of this pathway (Hubbard, 2018). In human skin cells and other mammalian cells, the JAK family typically consists of 4 membrane-associated Janus family kinases (JAKs), JAK1, JAK2, JAK3, and TYK2 which together constitute an important group of non-receptor tyrosine kinases. JAK-STAT pathway is activated upon ligand-mediated receptor multimerization which sets two adjacent JAKs into close proximity and facilitates *trans*-phosphorylation. Upon activation, JAKs phosphorylate signal transducer and activator of transcription proteins (STATs) and several other substrates, including both the receptors and the major non-receptor substrates (Xin et al., 2020). The STAT family comprises of 7 latent transcription factors (i.e., STAT1, STAT2, STAT3, STAT4, STAT5a, STAT5b, and STAT6) that typically shuttle between cytoplasm and nucleus in response to extracellular stimuli or through deregulated activation of other aberrant signaling events (Guanizo et al., 2018). This shuttling mechanism of STATs is strongly influenced by phosphorylation. Once phosphorylated, phosphotyrosine residues allow STATs to dimerized through their interaction with conserved SH2 domains (Szelag et al., 2015). A negative regulation of the JAK/STAT pathway, which shuts this pathway off, is ensured by an array of kinases, phosphatases (PTPs), cytokine receptors, suppressors of cytokine signaling (SOCS) proteins, and protein inhibitors of activated STATs (PIAS) (Bang, 2019). In melanoma cells, STAT proteins are frequently constitutively phosphorylated or constitutively activated in response to intracellular or extracellular stimuli or through unregulated activation of other signaling molecules (Rovida and Stecca, 2015). Phosphorylated STATs enter into the nucleus in the form of dimers and bind to specific regulatory sequences, thereby activating or inhibiting the transcription of specific target genes (Wingelhofer et al., 2018). For example, STAT1-mediated gene expression is linked with growth retardation, apoptotic resistance, and reduced angiogenesis (Mohassab et al., 2020). Conversely, the gene expression induced by STAT3 has been found to be associated with cell proliferation, apoptotic resistance, metastasis, angiogenesis, and suppression of immune response (Chai et al., 2016). Thus, melanoma cells display distinct gene expression profiles depending on the level of activation of individual STAT proteins (Lopez-Bergami et al., 2008). A recently conducted study has shown that the ratio of phosphorylated STAT1 to STAT3 may be a biomarker that can predict the progression of melanoma (Verhoeven et al., 2020). Interestingly, STAT proteins have been found to be altered in both melanoma cells and in the immune system of melanoma patients (Tucci et al., 2019). Thus the JAK/STAT pathway imparts a mechanism that is used by melanoma cells to transduce extracellular stimuli into specific transcriptional responses.

Several strategies have been used to inhibit the STAT3 pathway as a therapeutic approach for treating malignant melanoma (Lee et al., 2019). Direct STAT3 inhibitors can be categorized into different classes of compounds: peptides, peptidomimetics, peptide aptamers, small molecules, platinum complexes, siRNAs, and plant polyphenols (Lee et al., 2019; Thilakasiri et al., 2021). Each of these STAT3 inhibitors has merits and demerits. For example, while peptide-based inhibitors exhibit a high degree of specificity, they possess limited cell permeability and *in vivo* stability (Aftabizadeh et al., 2021). More recently, cell-permeable, STAT3 SH2 domain mimetics with promising anti-tumor effects have been designed (Lai et al., 2015; Du and Lovly, 2018; Franke et al., 2018). Similarly, siRNA specific for the SH2 domain of STAT3 have been designed that have shown promising antitumor effects in prostate cancer cells and patient-derived murine xenograft tumor models (Son et al., 2017). Certain platinum complexes have shown remarkable antitumor activity by their ability to inhibit STAT3 in a variety of tumor models (Lazarević and Rilak, 2017). Likewise, natural products (e.g., resveratrol, flavopiridol, indirubin, magnolol, piceatannol, parthenolide, EGCG, cucurbitacin Q, and curcumin) have been demonstrated to downregulate the activity of STAT3 in cancer cells (Arshad et al., 2020). Besides, other studies also provide a rationale for inhibiting STAT3 as a therapeutic target in cancer cells. However, the specificity and selectivity of STAT3 inhibitors and inhibitors of other STAT proteins should be properly validated in various biological systems before final recommendations on the clinical use of these inhibitors are put forward (Huang et al., 2020). Arguably, certain small molecule inhibitors of STAT3 (e.g., Static, STA-21, S32-M2001, S3I-201) have shown remarkable antitumor activity by their ability to induce apoptosis [52]. A novel small molecule S3I-201.1066, a structural derivative of S3I-201, has recently been shown to disrupt phosphotyrosine interactions at the STAT3 SH2 domain with a high degree of efficacy (Beebe et al., 2018). Likewise, LLL-3, an early generation STAT3 inhibitor, has shown similar antitumor activity in different tumor models. FLLL32, a structural analog of curcumin, specifically inhibits STAT3 while retaining STAT1 mediated signal transduction within melanoma and immune-sensitive cells (Paulraj et al., 2019). Treatment of different melanoma cells with FLLL32 triggers caspase-dependent apoptosis via inhibition of STAT3 (Paulraj et al., 2019) (Table 1).

**TABLE 1:** STAT3 inhibitors in melanoma.

Small molecule inhibitor(s)	Mechanism of action
Stattic, STA-21, S32-M2001, S3I-201	Antitumor activity by their ability to induce apoptosis *via* STAT3 inhibition
LLL-3, an early generation STAT3 inhibitor	Antitumor activity in different tumor models
FLLL32, a structural analog of curcumin	Inhibits STAT3 while retaining STAT1 mediated signal transduction within melanoma and immune sensitive cells, FLLL32 triggers caspase-dependent apoptosis via its inhibition of STAT3
Resveratrol, flavopiridol, indirubin, magnolol, picetannol, parthenolide, EGCG, curcubitacin Q and curcumin	Downregulate the activity of STAT3 in cancer cells
Peptide aptamers	Interacts with a dimerized form of STAT3 thereby causes inhibition of antiapoptotic proteins and promotes apoptosis in melanoma cells
S31–1757 and S31-201 micelle formulation	Inhibits STAT3 dimerization in both cellular and animal models of melanoma with less undesirable effects on normal cells
Novel platinum (IV) compounds (CPA-1, CPA-7)	Disrupts STAT3 signaling, thereby abrogates tumor-promoting activity of STAT3
C48	Promotes alkylation of STAT3 at cys468 thereby attenuating the accumulation of activated STAT3 in the nucleus to inhibit the growth of melanoma cells

### JAK/STAT Signaling Gastric Cancer

Gastric cancer (GC) is a heterogeneous malignancy and is one of the leading cancer-related mortalities across the world (Jan et al., 2021; Rah et al., 2021). The major risk factors contributing to gastric tumorigenesis are environmental factors, geographical location, lifestyle factors, age, and chronic infection with *Helicobacter pylori* (Chia and Tan, 2016; Jan et al., 2021; Karimkhani et al., 2017). Besides environmental and other risk factors, recently conducted whole genome sequencing and genome-wide association studies have led to the identification of novel genomic alterations/mutations which profoundly contribute to the pathogenesis of GC (Pectasides et al., 2018). Recent shreds of evidence suggest that 474 hotspot mutations in 41 genes were identified in gastric adenocarcinoma patients, among them *PIK3CA* harbored mutation ranks at the top followed by mutations in *TP53, APC, STK11, CTNNB1,* and *CDKN2A* genes. These genomic mutations lead to aberrant activation of several signaling molecules, such as vascular endothelial growth factor receptor (VEGFR) family, epidermal growth factor receptor family (ErbB) members, and various components of JAK/STAT and PI3K/Akt/mTOR pathway thereby contributes in the molecular pathogenesis of GC (Khanna et al., 2015; Yeh et al., 2016; Zhu et al., 2019).

Although, several drugs have been developed for the treatment of GC, the overall prognosis for GC patients is still dismal (Ajani et al., 2017; Matsuoka and Yashiro, 2018). Currently available standard treatments for GC are surgery, chemotherapy, and radiation therapy (Sitarz et al., 2018). The most commonly used therapeutic regimen is ECX (epirubicin, cisplatin, capecitabine), ECF (epirubicin, cisplatin, 5-FU), EOX (epirubicin, oxaliplatin, capecitabine), and EOF (epirubicin, oxaliplatin, 5-FU) (Charalampakis et al., 2018). The second line regimen for GC therapeutics is irinotecan, docetaxel, and paclitaxel. Since GC is a heterogeneous disease, advances in personalized medicine should be improved with each patient being treated individually based on various genetic and epigenetic alterations regulating different signaling pathways with targeted drugs (Bonelli et al., 2019). Targeted therapy identifies and kills cancer cells specifically while leaving healthy cells unharmed. Monoclonal antibodies (such as HER2, VEGFR, or EGFR), multikinase inhibitors, and immune checkpoint inhibitor therapy (such as CTLA-4 or PD-1/PD-L1 are mainly involved in targeted therapy that has shown to be beneficial in the treating metastatic and advanced stages of GC patients (Seebacher et al., 2019). RNA interference and use of antisense oligonucleotides (ASOs) involving the silencing of target proteins has been explored as one of the STAT-inhibitory approaches which are targeting the JAK/STAT signaling pathways (Szelag et al., 2015). Recently, the use of short palindromic repeats (CRISPR) and its related proteins (Cas-9) have been explored as genome editing tools to mute the JAK/STAT pathway components (Bousoik and Montazeri Aliabadi, 2018).

Primarily known for immune regulation and plays a critical role in hematological malignancies harboring V617F driver mutation, JAK/STAT signaling cascade contributes strongly to the pathogenesis of GC. Besides, frequent amplification of the chromosomal region harboring JAK2 has been identified in GC. The most studied member of JAK/STAT is in GC is STAT3. Phosphorylation at tyrosine-705 residue of STAT3 leads to the aberrant activation of STAT3 resulting in increased growth, angiogenesis, invasion, and metastasis (Giraud et al., 2012; Loh et al., 2019). Hyperactivation of STAT3 was found in a number of GC cell lines and its elevated expression in histological sections of GC patients was strongly associated with angiogenic factors such as VEGF and microvessel density formation thereby contributing to GC progression (Qi et al., 2020). Furthermore, STAT3 expression was analyzed in gastric specimens with respect to a member of the regenerating gene family (REG Iα) to study its role in inflammation-associated with GC. The study further analyzed that REG Iα expression was strongly correlated with the phospho-STAT3 expression in GC specimens, suggesting that REG Iα plays a crucial role in inflammation associated gastric tumorigenesis by promoting antiapoptosis mechanism in gastric mucosal cells. Additionally, cytokine-associated (IL-6/IL-11) dependent elevated expression of STAT3 contributes to the development of H. pylori-associated GC in gp130^757FF^ GC established mouse models. STAT3 is known to control the transcriptional epithelial-to-mesenchymal (EMT) regulators through G1 and G2/M cell-cycle progression, leading to the progression of metastasis in gastric malignancies (Azarnezhad and Mehdipour, 2017; Eissmann et al., 2019). STAT3 promotes angiogenesis in GC by stimulating the production of hypoxia-inducible factor (HIF)-1 and VEGF (He et al., 2019). Immunohistochemistry revealed that STAT3 expression was strongly associated with lymph node metastasis, TNM staging, and survival, suggesting that STAT3 could function as a predictive biomarker for poor prognosis in GC (Zhang et al., 2017). Furthermore, aberrant STAT3 signaling promotes tumorigenesis by deregulating the expression of genes including p21^WAF1/CIP2^, MYC, BCL-2, cyclin D1, BCL-_XL_, matrix metalloproteinase 1 (MMP1), MMP7, MMP9, and survivin which regulates cell proliferation and survival (Srivastava and DiGiovanni, 2016).

Besides, the JAK/STAT pathway which serves as a hub for a variety of signaling networks, exhibits a tremendous potential of crosstalk with other signaling pathways (Mullen and Gonzalez-Perez, 2016), thus acts as an appealing target cascade not only for hematological malignancies but also for other solid malignancy including gastric tumorigenesis. The currently used clinical trials involving targeted drugs mainly emphasize JAK family members as compared to those targeting STATs. Substantial effort has been made to develop STAT inhibitors, however, limited success has been achieved due to issues with bioavailability, *in vivo* efficiency, and selectivity (Shouse and Nikolaenko, 2019). Out of all STATs, STAT 3 has proven to be one of the promising targets for molecularly targeted treatment (27). The JAK/STAT signaling is activated in a substantial proportion of solid tumors and promotes to cancer cells aggressive characteristics, making it a promising target for novel therapies (Sabaawy et al., 2021). Several *in vitro* and *in vivo* studies have validated the potent part played by STAT3 in precancerous physiology of the stomach, implying that STAT3 could be used as a predictive marker for diagnosis of GC, thus inhibiting STAT3 activity with several inhibitory molecules might help to prevent cancer (Khanna et al., 2015) (Table 2). Taken together, JAK/STAT-associated signaling pathways play a multifaceted role in GC carcinogenesis and are ideal for the combinatorial targeted therapies which could be explored as a potential for the identification of novel biomarkers for GC treatment.

**TABLE 2:** JAK/STAT signaling inhibitors, target molecules, effects, and clinical indications in gastric carcinoma.

Inhibitor	Target	Effect	Indication (clinical trial phase)
Non-peptide small molecules			
Pravastatin	STAT1	downregulates IFN-γ levels	gastroesophageal cancer (phase IV)
Non-peptide small molecules			
pravastatin	STAT1	Phosphorylation inhibition of STAT1, decrease in IFNγ levels	gastroesophageal cancer (phase IV)
Natural products			
Vinorelbine	STAT3	Inhibition of STAT3 phosphorylation targets STAT3–tubulin interaction	gastric cancer (phase II)
Paclitaxel	STAT3	Prevents STAT3 phosphorylation, dissociation of STAT3 and, tubulin binding	Stomach cancer(phase I/II/III)
Tyrosine kinase inhibitors			
Sorafenib	JAK2, STAT3	Inhibits phosphorylation of STAT3	gastric cancer (phase I/II)
AZD148	JAK1, JAK2	Inhibition of STAT1, STAT3, STAT5, and STAT6 phosphorylation	gastric cancer (phase I)

### JAK/STAT Signaling in Esophageal Cancer

Esophageal cancer (EC) is among the top 10 ranked malignancies in terms of both incidence and mortality. Histopathologically two major types of EC are esophageal adenocarcinoma (EAC) and squamous cell carcinoma (ESCC). ESCC, on the other hand, accounts for almost 90% of all EC cases worldwide (Uhlenhopp et al., 2020). Regardless of recent breakthroughs in treatment tactics, the 5-year life expectancy runs from 30 to 50%, with over 70% of cases occurring in the male population (Sung et al., 2021). ESCC is highly aggressive and diagnosed at an advanced stage associated with distant lymph node metastasis. The majority of the patients with ESCC present with poor prognosis and high recurrence rates after surgery (Ohashi et al., 2015). Conventional treatment regimens including surgical intervention and radiotherapy have proven to be less effective in ESCC because of their high-grade invasive nature (Barzilai et al., 2019). Despite profound advancement in chemotherapeutic drugs, the overall survival time for the EC remains dismal and the management of disease outcome and eventually the quality of life is poor. Therefore targeted therapy is seemingly important for the treatment of EC. Signaling pathways have proven to be promising targets for targeted cancer therapies. Despite the signaling pathways being dysregulated at many levels, however, the critical junctures of signaling pathways that are frequently disrupted are receptors, transducer proteins, or transcriptional factors. Although a plethora of molecular signaling pathways plays a critical role in the EC pathogenesis, however, in the current review we mainly focus on JAK/STAT signaling in EC and its molecular targets for future therapeutics. Numerous studies have documented that the JAK-STAT pathway regulates molecular processes involved in EC progression including proliferation, survival, differentiation, and metastasis (Yang et al., 2020).

Aberrant activation of STAT3 contributes to esophageal cellular transformation, angiogenesis, epithelial to mesenchymal transition, and metastasis to distant sites. The association of the JAK/STAT pathway and prognosis of patients with ESCC was first confirmed by immunohistochemistry in 100 ESCC tumors and adjacent normal esophageal epithelia (Sugase et al., 2017). Studies suggested a significant correlation between phosphor-JAK1 and phosphor-STAT3 expression and prognosis of the disease in which an increased expression of *p*-JAK1 and p-STAT3 in primary ESCC tumors predicted the poor prognosis of ESCC patients (Zhao et al., 2021). Moreover, the expression levels of both proteins were found to be statistically significant with lymph node metastasis and TNM staging (Cheng et al., 2019). Therefore, STAT3 is a promising target for designing specific inhibitors for future therapeutics against EC.

The JAK/STAT pathway plays a critical role in the process of inflammation and EC progression (Pencik et al., 2016). In *in vitro* settings, AG490, a JAK2 inhibitor, was reported to decrease the inflammation and development of ESCC by preventing the constitutive stimulation of STAT3 (Liu et al., 2018). Cell cycle experiments further confirmed the STAT3 mediated regulation of cell cycle and proliferation in ESCC progression (Lu et al., 2019). Furthermore, STAT3 phosphorylation and COX-2 an important mediator of inflammation were highly expressed and consistently co-related with the ESCC cell lines (Cho et al., 2017). AG490 decreased the STAT3 activation and COX-2 protein levels significantly. STAT3 was also found to be involved in IL-6 mediated inflammation in ESCC cell lines (Liu et al., 2018).

Recently, a diterpene Cryptotanshinone (CTS), purified root extract from medicinal herb *Salvia miltiorrhiza Bunge* (DanShen) was employed *in vitro* and *in vivo* as a potent compound for the treatment of ESCC (Ji et al., 2019) (Table 3). In a dose-dependent experiment carried out in CAES17 and EC109 esophageal cancer cells, CTS substantially decreased the phosphorylation of STAT3 (Tyr705) and JAK2 expression level cells in response to IL-6 stimulation (Ji et al., 2019). Phlorizin, a member of the dihydrochalcone family derived from sweet tea is commonly used as a traditional medicine in China. Owing to having promising pharmacological importance, phlorizin inhibits esophageal malignant cell properties by activating apoptosis, autophagy and attenuating JAK2/STAT3 signaling (Jia et al., 2021). Together, these studies suggest that although STAT3 is a well-known mediator for tumor progression in numerous malignancies, however, it could be used to design specific pharmacological inhibitors derived from different sources for future therapeutics against EC.

**TABLE 3:** JAK/STAT signaling modulators, their targets, and mechanism in esophageal carcinoma/cells.

Compounds	Targets	Mechanism
Phlorizin	JAK2	Inhibits malignant properties of esophageal carcinoma cells by dephosphorylation of JAK2 and STAT3 proteins
	STAT3	
Stattic	STAT3	Inhibits dimerization of STAT3 by blocking tyrosine phosphorylation of SH2 domain of STAT3
Nimesulide	JAK	Reduces the JAK/STAT expression signaling by blocking cyclooxygenase-2 enzyme expression
	STAT	
Ellagic acid	STAT3	Inhibits both cytokine driven STAT3 and endogenous STAT3 signaling and promotes apoptosis to ESCC cells
NVP-BSK805	JAK2	Inhibits kinase activity of JAK2 by dephosphorylation
Cryptotanshinone	JAK2	Significant reduction of p-STAT3 (Tyr705) and *p*-JAK2 expression in ESCC cells
	STAT3	

### JAK/STAT Signaling in Hepatocellular Carcinoma

The most common type of primary heterogeneous malignancy affecting the liver is hepatocellular carcinoma (HCC) (Khemlina et al., 2017). HCC has gained the fifth rank in the list of most common cancers worldwide, and is responsible for the vast majority of cancer-related deaths (Bray et al., 2018). The most important factor that contributes to the development of HCC and HCC-related deaths is cirrhosis (Shiani et al., 2017). Nearly 80% of the population is developing HCC from chronic liver inflammation (Ghouri et al., 2017; Keenan et al., 2019). Besides, the high risk of hepatitis B virus (HBV) and exposure to aflatoxin-B1 (Kucukcakan and Hayrulai-Musliu, 2015; Irshad et al., 2017; Ivanov et al., 2017; Isaac and Burke, 2019) the other predominant molecular mechanisms responsible for the pathogenesis of HCC includes liver tissue damage which leads to cirrhosis and mutations in tumor suppressor and oncogenes (Alqahtani et al., 2019). These two mechanisms are associated with a dysregulation in the cell signaling pathway and lead to HCC (Gao JZ. et al., 2015). Although, various pathways such as wnt/β-catenin signaling pathway, PI3K/AKT/mTOR signaling pathway, receptor tyrosine kinase pathway, TGF-β signaling plays a critical role in the pathogenesis of HCC. However, JAK/STAT signaling equally contributes to the pathogenesis of HCC (Huang and Fu, 2015; Erkisa et al., 2021). In many human cancers including HCC, STAT3 is considered as a strong bona fide candidate promoting tumorigenesis (Khan AQ. et al., 2019). STAT3 activation as a transcriptional factor promotes a plethora of genes that contributes too many cancer hallmarks, thereby highlighting the tumorigenic role of STAT3 in HCC. STAT3 has a crucial role in G1 to S phase cell cycle progression by upregulating CCND1 expression. Additionally, STAT3 downregulates the expression of proapoptotic proteins TP53, Bax, Chop and upregulates the expression of antiapoptotic proteins Bcl-2, Bcl2-xl, Birc5, Mcl1, respectively in HCC cells. Many studies have reported the aberrant activation of JAK1 and STAT3 in the development of HCC which are the key members of the JAK/STAT signaling pathway and promote tumorigenesis (Hin Tang et al., 2020). It has also been shown that the JAK1 aberrantly phosphorylated STAT3 which results in cell proliferation, migration, invasion, and angiogenesis in HCC (Hin Tang et al., 2020). Aberrant activation of STAT3 in HCC is due to many growth factors and cytokines like TNF, IL-6, hepatocytes growth factor (HGF), and epidermal growth factor (EGF) family (Zhao et al., 2015; Ray et al., 2018). Abnormal release of cytokines stimulates JAKs, which eventually phosphorylated STAT3 at a critical tyrosine residue Tyr-705 or Ser-727 in HCC (Pinjari and Meerza, 2017). The activity of JAK and STAT3 is enhanced due to the overexpression of pro-inflammatory cytokines like IL-6, IL-10, IL-11, and TGF-α to regulate the tumor micro-environment, which eventually create the oncogenic conditions to inhibit apoptosis (Waters and Brooks, 2015; Ferrao et al., 2016; Yuan et al., 2017; Wu et al., 2018). Reports suggest that tumor aggression is associated with the activation of STAT3. Constitutive activation of STAT3 in HCC is also known to upregulate various miRNA’s including miR-21. Missense mutation of JAK1 has also been identified in HBV-infected HCC patients (Tezcan et al., 2019). With the increase in JAK1 mutation, there is an increase in phosphorylation of JAK1 and STAT3 without cytokine stimulation (Sims, 2020).

A number of pharmacological inhibitors can be used for the inhibition of the JAK/STAT signaling pathway in controlling HCC (Mansour et al., 2014; Poulou et al., 2015; Gao et al., 2015b; Duffy et al., 2017; Luo, 2017; Zhu et al., 2017; He et al., 2018; Hiraoka et al., 2019; Hin Tang et al., 2020; Pinter et al., 2021). SSI-1 (STAT induced STAT inhibitor-1) is the inhibitor for STAT3 activation. Akira et al. and Nakajima et al. reported that the mRNA expression of SSI-1 inhibitor was induced by interleukins like IL-4, IL-6, and G-CSF (Rautela and Huntington, 2017). Ruxolitinib is one of the specific JAK1/JAK2 inhibitors approved by the FDA against myelofibrosis. Although, ruxolitinib for HCC is still in the preclinical stage, however, recent studies suggest that ruxolitinib attenuates cell proliferation and colony-forming ability in HCC cells (Shimoda et al., 2020). Filgotinib is a selective inhibitor for JAK1 (Westhovens et al., 2017) in combination with ruxolitinib inhibits cytokines like IL-11, G-CSF with a mild increase in hemoglobin (Schwartz et al., 2017). Stattic, a small molecule inhibitor, abrogates STAT3 dimerization and translocation which eventually inhibits STAT3 activation in a phosphorylation-independent manner in many cancers. While Stattic augments apoptosis thereby reducing dose-dependent cell survival and invasiveness in HCC cell lines prior treated with radiations. Another small molecule inhibitor OPB-111077 when used singly against HCC had minimal toxicity issues; however, when given in combination with sorafenib, OPB-111077 is effective against HCC with an acceptable safety profile. Additionally, OPB-31121, another STAT3 inhibitor in clinical phase-I against advanced solid tumors had acceptable antitumor activity against HCC, however, due to its side effects on the peripheral nervous system; its usage should not be long-term. Napabucasin in combination with paclitaxel is in clinical trials against GC; however, Napabucasin was reported to inhibit tumor growth of HCC cells and xenograft. AZD9150, a siRNA-based drug used against B-cell lymphoma patients to target STAT3. In HCC the AZD9150 was tested and was found to have good efficacy and well tolerable safety profile. There are various compound/small-molecule inhibitors having an impact on JAK/STAT signaling in HCC are listed in Table 4. Collectively, these results suggest that JAK/STAT signaling is promising signaling to target in HCC. Although the clinical outcomes of these inhibitors is still in the infancy stage for HCC, however, the beneficial effects documented against other malignancies could underline the possible clinical efficacy against HCC.

**TABLE 4:** JAK/STAT signaling modulators, their targets, and mechanism in esophageal carcinoma/cells.

Compounds/small molecule inhibitors	Targets	Clinical status	Mechanism
WP1066	JAK2	Phase I	Inhibits JAK2 phosphorylation thereby attenuating cell migration and invasion by inhibiting MMPs
Pacritinib	JAK2	Pre-clinical	Pre-clinical studies showed that fibrotic areas of the mouse liver were effectively reduced by reducing CK18 biomarker
CTS	JAK2	Pre-clinical	In mouse models, CTS promotes apoptosis of tumor cells and helps to activate tumor-suppressive M1 cells
	STAT3		
Ruxolitinib	JAK1/2	Pre-clinical	Inhibits cell proliferation and colony-forming ability of HCC cells
Stattic	STAT3	Pre-clinical	Stattic inhibits tumor cell function in HCC such as cell proliferation and invasiveness
OPB-111077	STAT3	Phase I	Inhibits STAT3 in patients who are at an advanced stage of HCC and are not responding to sorafenib therapy
OPB-31121	STAT3	Phase I/II	Inhibits STAT3 in patients who are at an advanced stage of HCC and are not responding to sorafenib therapy
Napanucasin (BBI608)	STAT3	Phase Ib/II	Promotes antitumor activity in advanced HCC patients with prior systemic Sorafenib therapy
AZD9150	STAT3	Phase Ib/II	Promotes antitumor activity in advanced HCC patients with prior systemic Sorafenib therapy

### JAK/STAT Signaling in Head and Neck Squamous Cell Carcinoma

Head and neck squamous cell carcinoma (HNSCC) is mostly derived from mucosal epithelial of the buccal cavity (Sung et al., 2021). The consumption of alcohol or exposure to tobacco-derived carcinogens increases the burden of HNSCC with prior infection to the oncogenic strain of the human papillomavirus (HPV). While the presence of a proportion of pre-malignant lesions could advance towards invasive malignancy, however, majority of HNSCC patients are clinically diagnosed at late stages of malignancy. Therapeutic modalities for HNSCC are surgical resection followed by chemoradiotherapy (CRT). Despite the frequent mutations in key tumors suppressor genes such as NSD1, CDKN2A, TP53, NOTCH1, PIK3CA, TGFBR2, and FAT1, the dysregulation of associated signaling pathways also contribute to the development of pathophysiology of HNSCC. Aberrant activation of key signaling pathways which contributes in HNSCC are PI3K/Akt/mTOR pathway and STAT3 (Chen et al., 2021). The constitutive activation of the JAK/STAT pathway in response to several upstream signaling pathways, especially the TGF/EGFR pathway, has been associated with aberrant STAT3 activation in HNSCC (Gkouveris et al., 2016). In several types of HNSCC cells, JAK/STAT signaling may also be activated by the alpha-7 nicotinic receptor, IL-6, IL-10, and IL22 receptor (Gkouveris et al., 2016). There is compelling evidence that strongly suggests that persistent activation of JAK/STAT in HNSCC is associated with an increase in STAT3 tyrosine phosphorylation (Gutiérrez-Hoya and Soto-Cruz, 2020). The tumorigenic role of JAK/STAT signaling in HNSCC has been emphasized in a number of studies. JAK/STAT signaling is constitutively activated and plays a crucial role in regulating various cellular processes including cell proliferation, differentiation, and apoptosis in HNSCC (Groner and von Manstein, 2017). The aberrant increase in STAT3 tyrosine phosphorylation is believed to be a potent inducer of HNSCC progression and initiation. The oncogenic potential of JAK/STAT signaling is mainly dependent on the Tyr705 phosphorylation status whereas STAT3 serine phosphorylation still remains elusive (Morgan and Macdonald, 2020). The aberrant activation of JAK/STAT signaling and its association with constitutively expressed p-STAT3 at tyrosine/serine and total STAT3, Erk1/2, and cyclin D1 has been observed in oral squamous cell carcinoma (OSCC) cell lines (Gkouveris et al., 2016). Besides regulating cell proliferation and cell cycle progression, JAK/STAT signaling is also implicated in apoptosis, angiogenesis, immune evasion, as well as exerting effects on cancer stem cells. (Groner and von Manstein, 2017). The constitutive activation of JAK/STAT signaling is documented in diverse malignancies and is associated with the transactivation of several genes in malignant cells both *in vitro* and *in vivo* settings (Mali, 2015). Increasing STAT3 phosphorylation in primary HNC tumors and normal mucosa from HNC patients indicates early STAT3 activation in carcinogenesis (Aggarwal et al., 2021). Furthermore, a study has been performed and reported the increased STAT3 expression in smokeless tobacco-mediated HNC with minimal STAT3 in normal tissues (Sinevici and O’sullivan, 2016). Moreover, immunohistochemistry staining for phosphorylated STAT3 has also revealed the increased expression of activated STAT3 in human squamous cell cancer of the tongue correlated with poor prognosis in patients with HNC (Lee et al., 2017).

The role of cancer stem cells in HNC has been extensively studied making them an appealing target for further investigation. In addition to providing insight into how JAK/STAT functions, understanding its potential crosstalk with other molecules may lead to new treatment strategies for patients with HNSCC (Byeon et al., 2019). A number of studies in human tumors and HNC cell lines have identified JAK/STAT signaling pathway, specifically, STAT3 as a potential therapeutic target (Liang et al., 2020). A study by Kowshik et al. demonstrated that dietary supplementation of astaxanthin inhibited tumor progression by attenuating JAK/STAT signaling and its downstream target molecules including cyclin D1, MMP-2, -9, and VEGF in the HPV-induced tumor models (Kowshik et al., 2014). STAT3 decoy inhibitors inhibit STAT3 dimerization which is essential for translation into the nucleus to act as a transcriptional factor for a plethora of tumor survival genes. These inhibitors are taken in the form of injections and are used currently against the patients of HNSCC with a safe toxicity profiles. Another study by Ahn et al. showed Guggulsterone (GS) and other inhibitors (Table 5) decreased p-STAT3 expression in multiple myeloma and HNSCC cell lines (Siveen et al., 2014). Honokoil, a phytochemical derived from Magnolia Officinalis diminishes p-STAT3 tyr705, *p*-JAK tyr1007 as well as mRNA expression of respective genes in HNSCC cells. Leeman-Neill et al. also demonstrated growth inhibitory effects of guggulsterone in HNSCC preclinical models mediated by modulation of STAT3 signaling (Leeman-Neill et al., 2009). A natural triterpene, brusatol has been reported to act as a blocker for STAT3 and inhibits upstream kinases such as JAK1/2 thereby preventing the HNSCC progression. An artemisinin analog, dihydroartemisinin exhibits cytotoxicity potential and promotes apoptotic activity by diminishing activated JAK2/STAT3 expression in HNSCC cells. A significant association of smokeless tobacco consumption habits and accumulation of nuclear p-STAT3 was observed in clinical oral squamous cell carcinoma tissues (Zhou et al., 2016). Wang et al. suggested that OSCC cell proliferation and cell cycle regulation might be associated with the overexpression of JAK/STAT and cyclin D1 (Wang et al., 2019). Taken together, these findings collectively suggest that JAK/STAT signaling plays a critical role in HNSCC and is a promising signaling pathway to be targeted for drug development in HNSCC.

**TABLE 5:** JAK/STAT signaling modulators, their targets, and mechanism in head and neck squamous cell carcinoma (HNSCC)/cells.

Compounds/small molecule inhibitors	Targets	Mechanism
AG490	JAK2	Promotes *in vitro* efficacy against laryngeal carcinoma cells
Curcumin	JAK2	Promotes apoptosis, arrests HNSCC cells at G2/M phase of cell cycle, and augments tumor regression in animal models
FLLL12	JAK2	Inhibits cellular growth of HNSCC and promotes apoptosis by upregulation of pro-apoptotic proteins, inhibits Tyr705 phosphorylation of STAT3
	STAT3	
Stattic	STAT3	Stattic inhibits SH2 domain and decreases tumor growth in xenograft model of HNC
OPB-51602	STAT3	Binds SH2 domain of STAT3 thereby inhibits phosphorylation at Tyr705 and Ser727
AZD1480	STAT3	Inhibits cell proliferation and dephosphorylation of STAT3 in HNC cell lines
STAT3 decoy	STAT3	Inhibits STAT3 dimerization which is essential for translocation into the nucleus to act transcriptional factors for survival genes
Epigallocatechin Gallate (EGCG)	STAT3	Inhibits STAT3 action/function in HNSCC cells
Brusatol	JAK1/2, STAT3	Blocks upstream JAK1/2 and STAT3 activation in HNSCC cells
Dihydroartemisinin	JAK2 and STAT3	Exhibits antiproliferative and apoptotic activity by abrogating JAK2 and STAT3 activation in HNSCC cells
Honokoil	JAK and STAT3	Diminishes p-STAT3 tyr705, *p*-JAK tyr1007 as well as mRNA expression of respective genes in HNSCC cells

### JAK/STAT Signaling in Breast Cancer

Breast cancer (BC) is the most common heterogeneous malignancy and the second leading cause of cancer-associated mortalities in females. Thanks to worldwide screening for early diagnosis, BC has a better survival time compared with other gynecological malignancies. Apart from the genetic predisposition of BRCA1/2, PTEN and TP53 mutations in high-risk groups, the other genetic expression profile of estrogen receptor (ER), progesterone receptor (PR), and human epidermal growth factor-2 (HER-2) contributes to the pathogenesis and stratification of BC (Chen et al., 2020). Besides, mutations and epigenetic modifications in various critical genes, BC pathophysiology is also contributed by various signaling pathways. The major pathways that are dysregulated in BC are MAPK, PI3K/Akt/mTOR, and JAK/STAT signaling pathway. A significant number of growth factors and cytokines activate JAK/STAT signaling pathway (O’Shea et al., 2015). Various studies have shown that acquiring a functional mutation, and polymorphisms in various components of JAK/STAT pathway are responsible for the overactivation of the JAK/STAT pathway (Lokau and Garbers, 2019). Several mechanisms include autocrine/paracrine production of cytokines, activating mutations of different isomers of JAKs, its receptors, and various distant oncogenes constitutively stimulate this pathway which eventually further activates downstream STATs (Banerjee and Resat, 2016). The role of JAKs and STATs is not only restricted to inflammation, survival, and proliferation of cells but also implicated in a plethora of organ-associated tumorigenesis. Many reports in the recent past have shown that JAK/STAT has a significant role in the development of BC by acting either as oncogenes or tumor suppressors (Xing et al., 2021). In cancer, somatic mutations are rare in members of the JAK family. Regardless of this, it has already been described that JAK1, JAK2, and JAK3 have harbored somatic mutations in BC specimens (Kalimutho et al., 2015). Recent research exhibits the promising role of JAK2 and TYK2 in BC development. Studies have shown that the V617F point mutation causes constitutive activation of JAK2 in epithelial mammary cells, resulting in hyperactivation of STAT5 which eventually enhanced the proliferation of epithelial memory cells (Rani and Murphy, 2016; Velloso et al., 2017).

Other STAT proteins like STAT1, STAT3, STAT5, and STAT6 have also been associated with the progression, prediction, and prognosis of BC (Wang et al., 2018). In terms of physiological dependency perspective, STAT1 has been shown to act as a tumor suppressor or oncogene. Nearly 45% of androgen receptor (AR)-positive and 22% of AR-negative cases of BC showed evidence of reduced STAT1 levels in malignant cells compared to benign breast tissues (Haddon, 2018). On the contrary high expression levels of STAT1 are associated with therapeutic resistance and metastasis. This indicates that STAT1 acts as a tumor suppressor in menopausal ER^+^ BC, but STAT1 actively promotes tumor growth in ER^−^ tumors or ER^+^ tumors in premenopausal BC (Dong et al., 2020).

Solid tumors including BC usually have high activity of STAT3. Its aberrant turnover may advance invasion and metastasis, apart from regulating inflammatory reaction in breast tumorigenesis (Edsbäcker, 2019). It has been shown by various studies that STAT3 is perhaps the most often activated in primary BCs and is associated with tissue invasion and poor prognosis (Banerjee and Resat, 2016). The tumor microenvironment appears to get affected by the constitutive activation of STAT3 through the secretion of several cytokines, e.g., Interleukin-10, Interleukin-6, Interleukin1β, by cancer cells which activate noncancerous cells, tumor associated macrophages and T-helper (Th)-17 cells to release more cytokines, thereby contributing a positive feedback circle (Jeannin et al., 2018; Duan et al., 2020). This further elevates tumor cell growth and differentiation. IL-10 secretion directed by STAT3 in cancer cells also results in the arrest of antitumor immunity (McCaw et al., 2017).

In BC STAT5 like STAT3 is constitutively activated, but is deemed to be feebly oncogenic in mouse models of BC. STAT5 may possibly help in carcinogenesis, but it is not an acceptable proto-oncogene in BC according to current data available (Braicu et al., 2019; Recio et al., 2019). STAT6 regulates the balance of Th1 and Th2 cells, which promotes tumor growth by allowing tumor cells to evade immune responses. Depending on the cytokine secretion in the environment of T lymphocytes, T cells can differentiate either into Th1 or Th2 cells (Tough et al., 2020). Th1 cells helps in recognizing tumor antigens and eliciting an immune response, but Th2 cells are oncogenic, promote invasion and metastasis of tumors. STAT6 is required for T lymphocyte development in Th2 cells, which is mediated by the interleukin 4 (IL4) (Kim et al., 2018).

A large data shows the significance of the JAK/STAT pathway in various diseases of the immune system and in various cancers thereby drawing attention to therapeutic targets on key members of this pathway (Pectasides et al., 2018). In myeloproliferative diseases, JAK and STAT protein mutations have been comprehensively characterized and have been linked to hyperactivity of the JAK/STAT pathway which eventually leads to unrestricted cell proliferation (Vainchenker et al., 2018; Baldini et al., 2021). In myeloproliferative diseases, JAK and STAT protein mutations have been comprehensively characterized and have been linked to hyperactivity of the JAK/STAT pathway which eventually leads to unrestricted cell proliferation (Vainchenker et al., 2018; Baldini et al., 2021). Similar alterations have not been well studied in BC. However constitutive phosphorylation of STAT1, STAT3, and STAT5 is often found due to higher levels of cytokines and their respective receptors (Qin et al., 2019). Various studies have shown that the IL-6/JAK2/STAT3 pathway is upregulated in basal-like BC cells, and it has been stated by Marotta LL et al. that NBP-BSK805 and other inhibitors (Table 6) abrogates the growth of patient-derived breast tumor xenografts (Banerjee and Resat, 2016). Pyridone 6 another JAK inhibitor that was introduced in the early 2000s by Merck, has potential *in vitro* activity against JAK family members (Buchert et al., 2016). Ruxolitinib (Novartis) is another approved inhibitor for the treatment of myelofibrosis, which targets both JAK1 and JAK2 and is currently being used in a number of clinical studies (phase I, II, and III) in solid tumors including BC [173]. Apart from JAK inhibitors, (Winthrop, 2017) many molecules have been developed that primarily inhibit STAT3 and STAT5 e.g., a STAT3 inhibitor CJ1383 which shows good results in two BC cell lines having elevated expression of phosphorylated STAT3 (Roskoski, 2015; Bharadwaj et al., 2016; Beebe et al., 2018; Kim and Strober, 2018). IS3295 and FLL32 inhibitors have also shown promising results by inhibiting STAT5 in quite a few BC cell lines (Bharadwaj et al., 2016). Bcl-XL mediated expression and apoptosis of various tumor cells are regulated by STAT3 and STAT5. STAT3 and STAT5 are targeted by another anti-apoptotic Mcl-1 protein, a member of the Bcl-2 family (Maeda et al., 2018). Moreover, it was also demonstrated by Real *et al* that the overexpression of Bcl-2 mediated by STAT3 inhibited chemotherapy-induced apoptosis in BC cells (Liao et al., 2017), while another study by Masuda *et al* demonstrated that STAT3 inhibition improved the sensitivity of head and neck cancer cells to 5-Fluorouracil. Furthermore, survivin expression has also been shown to be induced by STAT3 (Aziz et al., 2021). In view of its anti-apoptotic activity and activation frequency in cancers, STAT3 could be used as an appealing therapeutic target in a number of cancers.

**TABLE 6:** JAK/STAT signaling modulators, their targets, and mechanism in breast cancer/cells.

Compounds/small molecule inhibitors	Targets	Mechanism
Pentagalloylglucose (PGG)	JAK1	Promotes dephosphorylation of JAK1 in MDA-MB-231 cancer cells and reduces tumor development in MDA-MB-231-induced xenograft tumor models
Methylsulfonylmethane	JAK2	Decreases phosphorylation of JAK2 in T47D and MCF-7 cells of breast cancer
Curcumin-BTP hybrids	STAT3	Represses STAT3 phosphorylation and nuclear translocation
BMA097	STAT3	Downregulation of STAT3 activated genes by dephosphorylation of the SH2 domain of SH2
Furanoditerpenes (Crispenes F and G)	STAT3	Inhibits STAT3 dimerization in MDA-MB-231 cancer cells which is essential for STAT3 activity
Alantolactone	STAT3	Reduces the pSTAT3 expression in MDA-MB-231 cancer cells
WMJ-8-B	STAT3	Increases SHP-1 driven reduction in MDA-MB-231 cancer cells by decreasing phosphorylation of STAT3
Galiellalactone	STAT3	Inhibits Tyr705 STAT3 phosphorylation

### JAK/STAT Signaling in Ovarian Cancer

Ovarian cancer (OC) is the most common cause of cancer-related deaths from gynecologic malignancies accounting for 4.3% of female deaths worldwide (Sung et al., 2021). The high mortality rate is due to the late-stage diagnosis, lack of effective early detection methods, and strong drive towards metastasis. Depending on the origin of tissue, OC has been classified into epithelial ovarian cancer (EOC), germ cell tumor, and stromal endocrine cell tumor. EOC, the most lethal of all gynecological malignancies, accounts for 90% with various other subtypes including serous, endometrioid, clear cell, and mucinous carcinoma (Cortez et al., 2018). Non-epithelial OC includes germ cell tumors and sex cord-stromal carcinoma accounting for 10% of OC (Maoz et al., 2020). Accumulating evidence indicates extensive crosstalk between various oncogenic signaling pathways such as PI3K/Akt, RAS/RAF/MEK/ERK, and JAK/STAT in a wide range of malignant neoplasms including OC (Yap et al., 2009). Interestingly, a number of studies have highlighted the significance of the JAK/STAT signaling pathway in the pathogenesis of OC. Aberrantly activated JAK/STAT signaling has been observed in various OC cell lines (OCC) as well as clinical tissue samples (Masoumi-Dehghi et al., 2020). The JAK/STAT signaling pathway plays a critical role in promoting cell proliferation, invasion, survival, stemness, angiogenesis, and chemo-resistance in OC (Liang et al., 2020) Figure 3. The critical role of JAK/STAT in enhancing tumor progression and survival is well established. Numerous studies have indicated that the aberrant activation of STATs strongly influences the expression of Bcl-2 (Groner and von Manstein, 2017). In addition, an increase in the expression of cyclin D1 and c-Myc has also been correlated with the constitutive activation of STAT3 thereby contributing to malignant transformations in human OC (Poli and Camporeale, 2015). Several studies have demonstrated the critical role of STAT3 in the migration and invasion of OC. Increased expression of pY-STAT3 in association with loss of protein inhibitor of activated STAT3 (PIAS3) resulted in the progression of high-grade serous carcinoma (Wu et al., 2020). The PKM2–STAT3/NF-κB axis activated by AKT2 enhanced the migratory and invasive capabilities of EOC cells. VEGF and HIF-1α, the two critical molecules in angiogenesis play a pivotal role in angiogenesis (Wu et al., 2020). Activated STATs (STAT3 and STAT5) have been found to regulate the expression of VEGF by directly binding to its promoter, thereby strengthening VEGF expression and tumor angiogenesis (Wu et al., 2020). In comparison with benign and normal tissues, immunohistochemistry showed increased expression of pY-STAT3, pY-STAT5, and VEGF in patient-derived ovarian epithelial carcinoma tissues (Wu et al., 2020). Also, immunohistochemical analysis revealed an increase in the nuclear expression of pY-STAT3 and HIF1α in ovarian clear cell carcinoma (Wu et al., 2020). IL-6 is known to induce transcription of VEGF and activates expression of downstream HIF-1α *via* STAT3. The upregulation of IL-6, STAT3, HIF-1α observed in ovarian clear cell cancer samples indicates an IL-6/STAT3/HIF1α/VEGF autocrine activation loop in EOC thereby facilitating tumor angiogenesis (Tawara et al., 2019). Recent advances in understanding the cellular signaling pathways have led to the development of several strategies and targets for various cancers including ovarian cancer (Wen et al., 2020). Despite chemotherapy being a crucial part of OC treatment, a significant number of patients develop chemoresistance. In addition, to killing cancer cells, chemotherapeutic intervention can also have cytotoxic effects on adjacent normal tissues and cells as well (Norouzi-Barough et al., 2018). To date, various alternate approaches have been carried out to inhibit JAK/STAT signaling including small molecules derived from natural sources, synthetic molecules, and antisense oligonucleotides (Yaswen et al., 2015). In preclinical or clinical trials, a number of natural and synthetic agents targeting JAK/STAT in OC are being explored (Bose et al., 2017, 2020). Resveratrol derived from red grapes and berries is known to possess anti-cancer activity in various cancers, including OC. Another study demonstrated that resveratrol significantly inhibited cell proliferation and induced apoptosis in OC patients (Rauf et al., 2018). Also, resveratrol inhibits growth, increases apoptosis and autophagy activity in OC cells by suppressing the STAT3 signaling pathway (Rauf et al., 2018). Curcumin (diferuloylmethane) is known to suppress STAT3 activation, resulting in decreased cell viability in OC cells (Perrone et al., 2015). Curcumin not only inhibits characteristics of OC through STATs but also blocks another signaling, including PI3K/Akt and NF-kB pathway (Perrone et al., 2015). Similar to curcumin and resveratrol, Corosolic acid, a potent STAT3 inhibitor, abrogates STAT3 activity resulting in the suppression of cell growth in OCC. In OC cells, Corosolic acid is also known to enhance the cytotoxicity of various chemotherapeutic drugs *via* inhibition of STAT3 activation (Willenbacher et al., 2019). The rapid metabolism or delivery systems and low bioavailability of natural compounds are some of the major concerns. Hence, the designing of corresponding analogs of natural compounds or synthesizing other novel small molecules that can abrogate JAK/STAT signaling has received great attention (ElNaggar et al., 2016). Curcumin analog diarylidenylpiperidone (DAP)-based synthetic compound, HO-3867 is known to block STAT3 activation by directly binding to the STAT3 DNA-binding domain. Treatment of human ovarian cancer cells with HO-3867 strongly decreased their migration (Liang et al., 2020). In a similar manner to HO-3867, DAP compound derivatives, HO-4200 and H-4318, also inhibit STAT3 by interacting with its DNA-binding domain (Liang et al., 2020). A study by El Naggar et al. demonstrated that HO-4200 and H-4318 significantly decreased the expression of STAT3 target proteins cyclin D1/D2, Bcl-xl, Bcl-2, survivin, and c-myc in cisplatin-resistant OC cell line suggesting cell survival inhibition and induction of apoptosis (ElNaggar et al., 2016). Also, HO-4200/H-4318 inhibited expression of VEGF, decreased migration, and invasion activity of OC cell line (ElNaggar et al., 2016). Besides DAP compounds, another STAT3 inhibitor, LC28 targets the DNA-binding domain of STAT3 (Liang et al., 2020). Huang et al. demonstrated that LC28 significantly blocked STAT3 interaction with DNA to inhibit the growth of cisplatin-resistant OC cells (Huang et al., 2018). Several other inhibitors of JAK/STAT signaling such as WP1066154, AZD1480153, MLS-2384155, and SD-102945 are also known for their anti-tumor property in OC models (Liang et al., 2020). Various upstream kinases or diverse cytokines, including IL-6, EGFR, and Src are known to activate JAK/STAT signaling pathway and are attractive strategies to abrogate JAK/STAT signaling (Wee and Wang, 2017; Gutiérrez-Hoya and Soto-Cruz, 2020). The decrease in the expression of the STAT3 by EGFR inhibitors such as Cetuximab, lapatinib, Erlotinib, and Gefitinib have shown minimal clinical activity in patients treated for OC (Seshacharyulu et al., 2012). Src, a cell membrane-associated non-receptor tyrosine kinase is known to activate STAT3 pathway and plays a decisive role in cell proliferation differentiation and migration of tumor cells. Src inhibitor, Dasatinib has shown minimal efficacy in patients with recurrent epithelial ovarian cancer (Zhang and Yu, 2012). However, combinatorial study has revealed the synergistic effect of dasatinib and paclitaxel on the inhibition of growth of ovarian granulosa cell tumor cells. IL-6, one of the critical cytokines that recruits gp130, forms IL-6/IL-6R/GP130 complex to activate STAT3 (Roze et al., 2021). The IL-6/gp130/STAT3 signaling axis is frequently aberrant in many tumors. Guo et al. demonstrated that a Monoclonal anti-IL-6 antibody, siltuximab significantly decreased the expression of STAT3 by inhibiting IL-6-induced STAT3 activation in OC cells (Liang et al., 2020). Another study by Coward et al. showed a significant decrease in nuclear expression of pY-STAT3 expression in intraperitoneal EOC xenograft (Wu et al., 2020) (Table 7). Collectively, these findings suggest that targeting JAK/STAT and an associated pathway is the ideal strategy for the future development of anticancer therapeutic lead molecules against OC.

**FIGURE 3:**
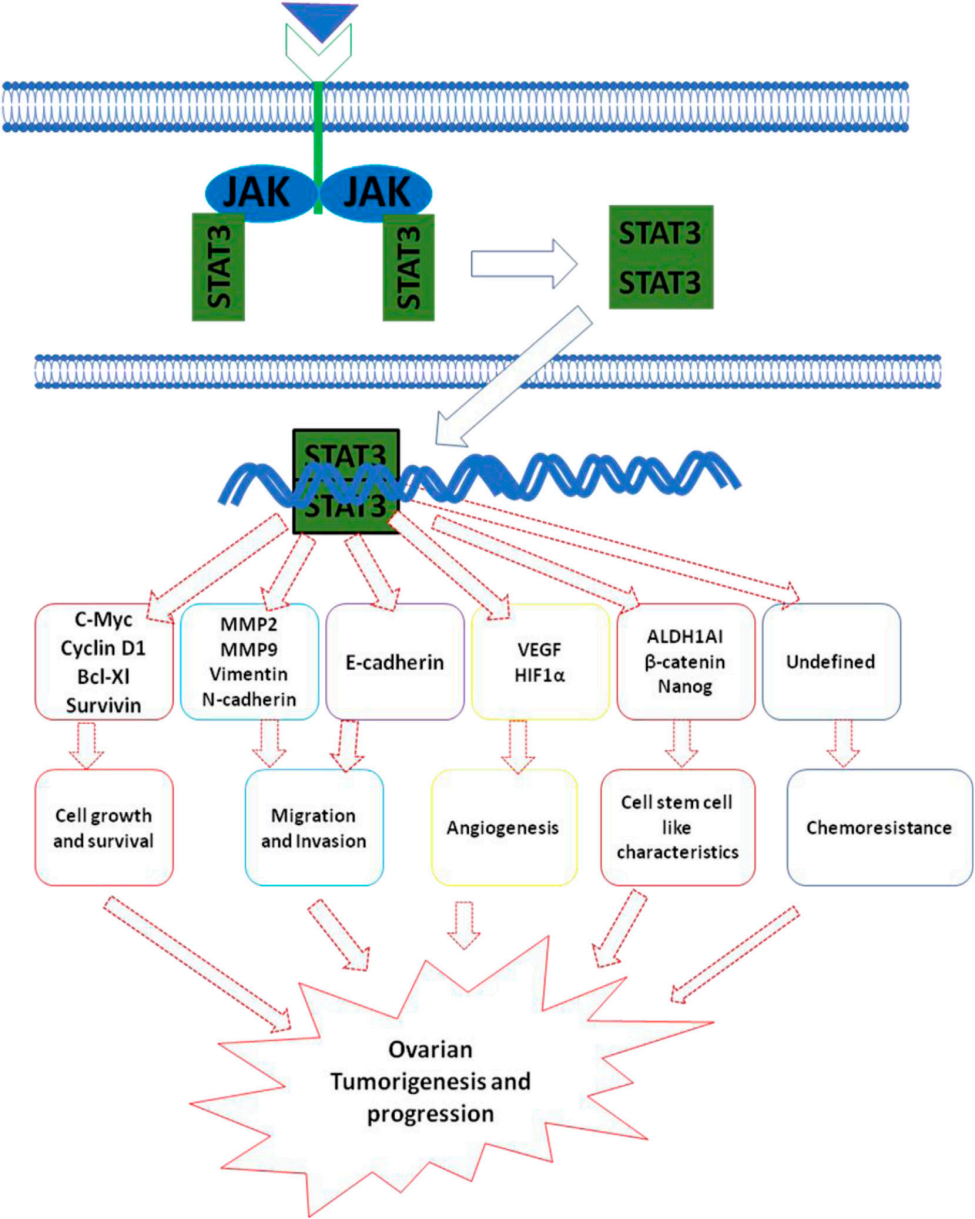
JAK/STAT signaling pathways its downstream mediators and mechanism associated with ovarian cancer.

**TABLE 7:** JAK/STAT signaling modulators, their targets, and mechanism in ovarian cancer/cells.

Compounds/small molecule inhibitors	Targets	Mechanism
AG490	JAK2	Reduces JAK2 phosphorylation in ovarian cancer cells
MLS-2384	JAK/Src	Promotes apoptosis and decreases cellular growth of ovarian cells by inhibiting JAK2 phosphorylation
Ruxolitinib	JAK2	Inhibits JAK2 phosphorylation
Resveratrol	STAT3	Suppresses tumor growth, cell proliferation and promotes apoptosis of ovarian cancer cells by targeting STAT3 phosphorylation
Diferuloylmethane	STAT3	Reduces STAT3 phosphorylation in ovarian cancer cells
Corosolic acid	STAT3	Decreases STAT3 expression by inhibiting M2 polarization of macrophages in the ovarian tumor microenvironment
Diarylidenylpiperidone (DAP)	STAT3	Inhibits activation of STAT3 by reducing phosphorylation

## Conclusion

JAK/STAT is one of the versatile signaling pathways which has been extensively studied for its crucial role in tumor progression. The potential crosstalk of JAK/STAT with multiple alternative pathways has made it a promising target for the development of new lead molecules. Aberrant activation of JAK/STAT signaling has been frequently observed in a wide range of malignant neoplasms. A number of studies have provided compelling evidence that inhibition of the JAK/STAT pathway provides significant therapeutic benefits. Several JAK/STAT inhibitors (natural as well as synthetic) have been found to modulate the expression of molecules involved in the JAK/STAT signaling network. Preclinical studies in cancer models have shown the effect of various inhibitors of JAK/STAT signaling which resulted in inhibition of cellular proliferation and tumor progression.

Thus, JAK/STAT signaling appears to be an important target with the potential for a high therapeutic index. Although it takes more than two decades to get approval from FDA to target JAK/STAT signaling. In the coming future, we expect more specific compounds with the least deleterious effects on normal cells which target particularly the kinase activity of JAK/STAT mediators to attenuate its amplification in malignancies. However, the big question is whether next-generation compounds/inhibitors will be able to attenuate the functional part of mutated JAKs and leave wild-type JAKs unaffected. This approach of inhibition could decrease the deleterious effects of compounds/inhibitors on normal cells and will improve the drug efficacy. Besides various preclinical and experimental settings, effective STAT inhibitors in clinical settings are still a dream to come true. Although targeting transcriptional factors is not common, however, this approach will lead to more specific inhibition and in the coming future could be a promising therapeutic approach against JAK/STAT signaling in solid malignancies. However, it is important to validate the mechanism of action of small molecule inhibitors (SMIs) of the JAK-STAT pathway before conclusions can be drawn about their clinical use. The hope is to administer SMIs of JAK-STAT pathway with minimum risk to human subjects and that these inhibitors will produce a significant therapeutic effect against the solid malignancies.
